# Post-transfusion Malaria in Morocco and Non-endemic African Countries: A Systematic Review of Reported Cases

**DOI:** 10.7759/cureus.104289

**Published:** 2026-02-26

**Authors:** Mouad Harandou, Mohammed Amine Nejjari, Mehdi Naciri, Abir Yahyaoui, Aziza Hami

**Affiliations:** 1 Laboratory of Parasitology-Mycology, Department of Central Laboratory, Faculty of Medicine, Mohammed VI University Hospital, Mohammed First University, Oujda, MAR

**Keywords:** blood transfusion safety, malaria-free countries, plasmodium falciparum, post-transfusion malaria, transfusion-transmitted malaria

## Abstract

Malaria remains a major global health problem, particularly in Africa. Although transmission is primarily vector-borne, Plasmodium can also be transmitted through blood transfusion, resulting in post-transfusion malaria (PTM). PTM is typically linked to asymptomatic parasitemia in donors and, while uncommon, may be rapidly progressive and potentially fatal. We conducted a systematic review to summarize published PTM cases in African countries considered non-endemic and to identify risk factors and prevention gaps relevant to transfusion services. We searched PubMed/MEDLINE, Google Scholar, and gray literature (including World Health Organization (WHO) documents, national transfusion resources, theses, and conference proceedings) in English and French with no date restrictions; the last search was 30 December 2025. We included case reports, case series, and abstracts with extractable case-level data describing PTM in non-endemic African countries, requiring laboratory confirmation of Plasmodium infection in the recipient and epidemiologic support for transfusion as the most plausible route. Reports suggesting non-transfusion transmission, lacking laboratory confirmation, or providing insufficient evidence implicating transfusion were excluded. Data were extracted using a standardized form, and case-series quality was appraised using the Joanna Briggs Institute (JBI) Critical Appraisal Checklist. Across all sources, 20 PTM cases were identified, but only six were described in sufficient detail for comprehensive extraction. Most detailed cases involved immunocompromised or medically complex recipients. Plasmodium falciparum predominated among fully described cases, consistent with prior reports, although Plasmodium malariae was also documented. Fever occurring days after transfusion was the most common presentation, and diagnosis was typically established by peripheral blood smear. When reported, investigations suggested donor exposure through travel or origin from endemic areas, highlighting the role of asymptomatic carriage and potential gaps in donor risk assessment. Countries without retrievable case-level reports (e.g., Cabo Verde, Lesotho, Seychelles, Egypt, and Libya) may have no accessible published cases, and this does not necessarily mean PTM is absent. PTM remains a rare but clinically important event in non-endemic African countries, with disproportionate risk in vulnerable recipients and potential for diagnostic delay. Strengthening hemovigilance, maintaining clinical suspicion for fever after transfusion, and refining donor risk assessment, potentially including selective testing strategies used in some non-endemic settings, may further reduce residual risk.

## Introduction and background

Malaria is an infectious disease caused by Plasmodium parasites [1]. It is a major global health problem, particularly in endemic African countries, according to the World Health Organization [2]. Five Plasmodium species are known to infect humans: Plasmodium falciparum, Plasmodium vivax, Plasmodium malariae, Plasmodium ovale, and Plasmodium knowlesi [3]. Transmission is primarily vector-borne through the bite of an infected female Anopheles mosquito, which introduces parasites into the bloodstream [4]. However, malaria can also be transmitted through blood transfusion, creating a potential route of spread, especially in non-endemic regions, where clinical suspicion and surveillance may be less robust than in endemic regions. Post-transfusion malaria (PTM) is most often related to asymptomatic parasitemia in donors and, although uncommon, may be rapidly progressive and potentially fatal [1,4]. These considerations underscore the need for strengthened vigilance and quality assurance across the transfusion chain, from donor screening to recipient follow-up. However, the available evidence in non-endemic African countries remains fragmented and largely limited to isolated reports, with no consolidated synthesis of recipient/donor features, diagnostic delays, and outcomes to inform transfusion policies. We conducted a systematic review to summarize published cases of PTM and to identify risk factors and prevention gaps relevant to transfusion services in non-endemic countries. Through this review, we aim to describe the occurrence of PTM in non-endemic African countries, focusing on recipient characteristics, donor origin, diagnostic delays, and clinical outcomes.

## Review

Materials and methods

Inclusion Criteria

We included case reports, case series, and abstracts with extractable case-level data describing PTM in African countries considered non-endemic (malaria-free) at the time of the study, with laboratory confirmation of Plasmodium infection in the recipient and clinical and epidemiologic assessment supporting transfusion as the most plausible route.

Exclusion Criteria

We excluded non-transfusion transmission cases (mosquito-borne, congenital, transplant-related, blood exposure); reports without laboratory confirmation in the recipient; and reports without sufficient information to support transfusion as the most likely route.

Planned Groupings for Synthesis

Cases were grouped by country and described according to year of occurrence, Plasmodium species, implicated blood product, and the recipient’s risk context (e.g., dialysis, transplantation, malignancy, or immunosuppression).

Information Sources

We searched and screened reference lists of included reports from PubMed/MEDLINE, Google Scholar, and Gray literature: World Health Organization (WHO) documents, national blood transfusion, theses, and conference proceedings accessible online. Last search date for all sources was December 30, 2025.

Search Strategy

PubMed search combined MeSH and free-text terms: ("malariae"[Mesh] OR"Malaria"[Mesh] OR malaria[tiab] OR plasmodium[tiab]) AND ("Blood Transfusion"[Mesh] OR transfusion[tiab] OR "transfusion transmitted"[tiab] OR "transfusion-transmitted"[tiab] OR “post-transfusion[tiab]”) AND ("Africa"[Mesh] OR Africa[tiab] OR Morocco[tiab] OR Algeria[tiab] OR Tunisia[tiab] OR Libya[tiab] OR Mauritania[tiab] OR "Cabo Verde"[tiab] OR Lesotho[tiab] OR Mauritius[tiab] OR Seychelles[tiab]). No filters were applied.

Data Collection Process

We extracted data using a standardized form. When key variables were unclear, we recorded them as not reported. We collected data on recipient outcomes and diagnostic timing. We also extracted laboratory confirmation methods and parasitemia levels when reported. Recipient-level variables included age, sex, transfusion indication, and relevant comorbidities. Transfusion-related details comprised the blood component type, number of units transfused. In addition, we recorded the identified Plasmodium species, donor origin, any travel or residence in endemic areas, and donor testing results. Finally, we documented key report characteristics, including publication year, country, and publication type.

Bias Assessment

Because the evidence base consisted largely of case reports, case series, and abstracts, we performed a structured quality appraisal rather than a traditional risk-of-bias assessment used for comparative studies. We applied the Joanna Briggs Institute (JBI) Critical Appraisal Checklist appropriate to each study design (case reports and case series), rating each item as yes, no, unclear, or not applicable (Appendices A-B). The appraisal was used to summarize reporting completeness and potential sources of bias (e.g., incomplete transfusion details, inconsistent donor assessment, and variable exclusion of alternative transmission routes) and to support cautious interpretation of the synthesis; studies were not excluded based on appraisal results.

**Figure 1:**
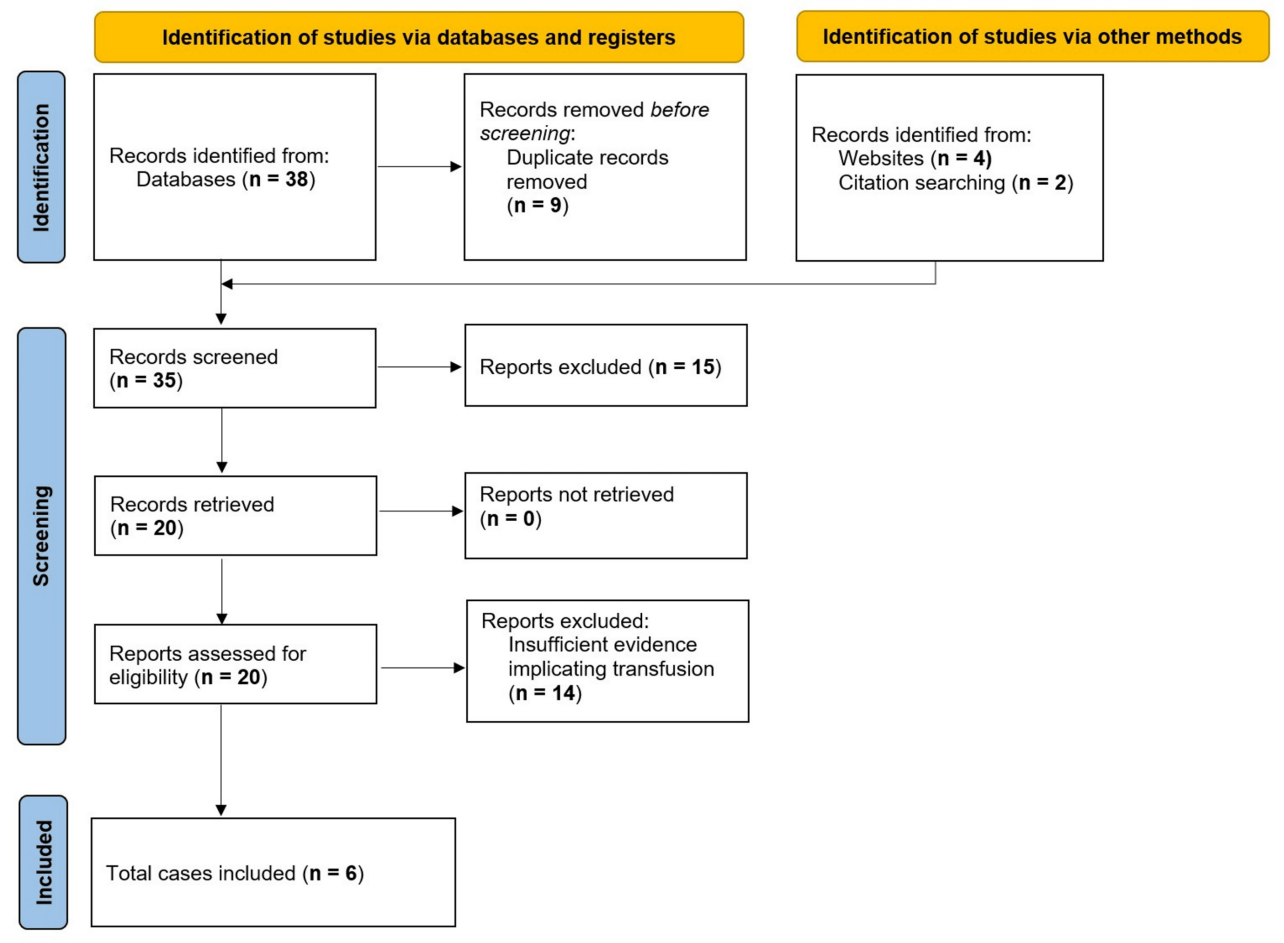
PRISMA flow diagram illustrating the literature search and study selection. PRISMA, Preferred Reporting Items for Systematic Reviews and Meta-Analyses

Figure 1 summarizes our literature search and study selection process.

Results

Our review, across all sources, revealed a total of 20 reported cases; only six were described in detail. Across Moroccan reports, three transfusion-associated malaria cases were identified, although several key details were not reported. In 2024, a 45-year-old woman with end-stage renal disease on hemodialysis was transfused for anemia and developed fever, fatigue, and splenomegaly approximately two weeks after red blood cell (RBC) transfusion; malaria was confirmed on peripheral blood smear as P. falciparum. She was treated with oral artemether-lumefantrine and improved rapidly, while the implicated donor (a male military donor) had recently returned from a tropical mission, and the donor's blood also tested positive for P. falciparum (donor treatment was not stated) [5]. In 2017, a 57-year-old hemodialysis patient with hypertension and goiter with hypothyroidism presented approximately three months after receiving two RBC units with fever, stupor, hepatomegaly, and splenomegaly. A blood smear showed P. malariae with an estimated 1% parasitemia. She received seven days of antimalarial therapy, followed by seven days of mefloquine, with a favorable outcome and negative follow-up smears. The 37-year-old male donor had traveled to Equatorial Guinea; however, donor testing and treatment were not reported [6].

In 2011, an 18-month-old child with acute lymphoblastic leukemia who received 20 blood products over 28 days was classified as post-transfusion P. falciparum malaria linked to an implicated RBC donation; however, clinical presentation, treatment, and outcome were not detailed. Donor serology was positive (indirect immunofluorescence (IFI): 1/160), but the donor could not be contacted [7]. A second 2011 case was identified only through citation in a secondary article (the primary report could not be retrieved), and several details were not reported. This case involved a 65-year-old French citizen who had traveled to Morocco, but the transfusion was performed in France for gastrointestinal bleeding. He received four RBC units from four different donors, including one of Gabonese origin. He subsequently developed fever and chills, and P. falciparum malaria was confirmed on smear and thick film with 12% parasitemia; despite treatment (not specified), he died 10 days later [8]. Because the transfusion occurred in France, we did not include this case among PTM cases occurring in Morocco (Table 1).

**Table 1:** Summary of transfusion-related malaria cases reported in Morocco. F, female; M, male; yo, years old; HD, hemodialysis; ESRD, end-stage renal disease; HTN, hypertension; ALL, acute lymphoblastic leukemia; GI, gastrointestinal; RBC, red blood cells; NR, not reported

Year/Reference	Recipient	Post-transfusion symptoms	Donor	Transfused blood product	Species/Confirmation	Outcome	Donor tests
2024 [5]	F/45 yo ESRD on HD, transfused for anemia	Fever, fatigue, splenomegaly	M/NR Military mission in tropical regions	RBC	P. falciparum smear	Clinical recovery after treatment	Donor blood positive for P. falciparum
2017 [6]	F/57 yo HD, HTN, hypothyroidism	Fever, stupor, hepatomegaly, splenomegaly	M/37 y, Moroccan, travels to Equatorial Guinea	NR	P. malariae smear, parasitemia (1%)	Clinical recovery after treatment	NR
2011 [7]	NR/18 months-old ALL	NR	NR/33 yo Origin NR Exposure NR	20 blood products	P. falciparum Smear, thick smear	NR	Donor serology positive

In Algeria, two transfusion-associated malaria cases were identified. In southern Algeria (Timimoune, Adrar Province), a transfusion-associated malaria case was reported in 2007. A 38-year-old woman hospitalized for anemia received a blood transfusion from an asymptomatic donor and was subsequently diagnosed with P. malariae infection. An epidemiologic investigation, together with parasitological and serologic testing of the donor, supported and confirmed that this episode was transfusion-transmitted malaria [9]. Another Algerian report from 1919 described a recipient who already had severe malaria (*pernicious malaria*) after exposure in Salonica and was hospitalized because of it. A subsequent malaria episode occurred in the donor approximately 15 days after donation, and the author interpreted this as accidental transfer during the transfusion procedure rather than newly transmitted malaria in the recipient. We, therefore, chose not to include this case as PTM (Table 2) [10].

**Table 2:** Summary of transfusion-related malaria cases reported in other non-endemic African countries. F, female; M, male; RBC, red blood cells; NR, not reported

Year/Country/Reference	Recipient	Post-transfusion symptoms	Donor	Transfused blood product	Species/Confirmation	Outcome	Donor tests
2007 Algeria [9]	F/38 y, transfused for anemia	NR	Asymptomatic	NR	P. malariae	NR	Parasitological and serological investigations confirmed the link.
2016 Tunisia [11]	M/27 y, Allograft recipient, transfused during hospitalization	Fever 15 days post-transfusion	African donor from Côte d’Ivoire, in Tunisia for 2 months	RBC and platelet units	P. falciparum thick film, parasitemia (20%)	Clinical recovery after treatment	Real-time PCR was positive for P. falciparum.
1962 Mauritius [12]	NR/30 y Euro-African origin Road-traffic trauma transfused on admission	Fever, then coma	Two donors. One of them, 49-y goat-keeper, of Indian origin	Two pints of blood from two different donors	P. falciparum thin films, parasitemia 15%	Clinical recovery after treatment	One donor was followed and later found to have minimal parasitemia.

In Tunisia, 14 transfusion-associated malaria cases were identified. The first case, in 2016, belongs to a 27-year-old man who had never traveled outside the country, who developed highly probable PTM after receiving red blood cell and platelet transfusions during hospitalization as an allograft recipient. His course was marked by antibiotic-refractory fever beginning 15 days after transfusion. On day 11 of fever, thick film and peripheral blood smear demonstrated P. falciparum trophozoites with high parasitemia (20%). He responded favorably to quinine. The epidemiologic investigation implicated an African donor from Côte d’Ivoire who had been in Tunisia for approximately two months, although initial donor testing was negative, real-time PCR was positive for P. falciparum, supporting transfusion-transmitted malaria (Table 2) [11].

It has also been reported that 11 cases were declared to the DSSB (Directorate of Primary Health Care) in Tunisia, with two additional presumed cases later noted: one at Farhat Hached University Hospital (Sousse) and another at Fattouma Bourguiba University Hospital (Monastir) [13]. In these two cases, the donors were African students, suggesting a potential reservoir of asymptomatic parasitemia among donors. However, the published reports did not provide sufficient detail for reliable data extraction (e.g., incomplete information on recipient characteristics, transfused component and timing, diagnostic confirmation, donor screening, and outcomes). Therefore, most of these cases were excluded from our tables and analysis and were not considered further in this study; only one case was discussed in detail (Table 2).

In Mauritius, only one case of transfusion-associated malaria was identified. Verdrager described a probable transfusion-transmitted Plasmodium falciparum infection identified during the malaria eradication program. A 30-year-old European assistant driver injured in a road accident received two pints of blood from two donors on admission and later developed fever, followed weeks afterward by coma. A blood film demonstrated P. falciparum with 15% infected erythrocytes and crescents, and he improved after antimalarial therapy. The episode was considered most consistent with post-transfusion infection; subsequent follow-up identified one donor with very low parasitemia months after donation (Table 2) [12].

In the remaining countries reviewed (Cabo Verde, Lesotho, Seychelles, Egypt, and Libya), we did not identify any published, case-level reports of PTM in the accessible sources searched. Accordingly, no cases from these countries could be included in our synthesis.

Discussion

Overview

Malaria is a parasitic infection caused by Plasmodium species [1]. Five Plasmodium species commonly infect humans: P. falciparum, P. malariae, P. vivax, P. ovale, and P. knowlesi [14,15]. P. falciparum is the most frequent cause of malaria in Africa [16]. P. malariae occurs in Asia, South America, and Africa and accounts for a small proportion of cases [17]. P. ovale is found mainly in Africa and the Western Pacific. P. knowlesi is primarily reported in Asia and is only rarely reported in African countries [15,18].

According to the WHO, global malaria case incidence increased in 2024 compared with 2023, driven largely by rises in several countries [17]. Malaria incidence varies substantially across populations; groups at higher risk include young children (particularly those who are immunocompromised), pregnant individuals, people living with HIV, and immigrants from endemic settings [2]. Multiple factors contribute to higher malaria incidence, especially in African countries, including low-socioeconomic conditions and limited access to prevention and healthcare services, with malaria often more common in rural areas [19,20]. Childhood malnutrition is also associated with increased mortality [21]. Transmission is influenced by climate, increasing with warmer temperatures and humidity, and typically peaking during the rainy season [2].

Vector-borne transmission remains the predominant route; however, malaria can also be transmitted through blood transfusion and tissue transplantation [4]. PTM is rare in non-endemic countries [22]. In the United States, 11 transfusion-transmitted malaria cases were reported from 1982 to 2022 [23-25]. Consistent with this low frequency, our review identified at most 20 cases from the accessible literature over the past six decades, of which only six were fully described. P. falciparum is frequently implicated in transfusion-transmitted malaria. However, some reports suggest a different distribution: in a systematic review by Verra et al., nearly half of transfusion-transmitted malaria cases in non-endemic areas were attributed to P. malariae and P. vivax [23]. In our review, four of six cases involved P. falciparum, whereas two of six were linked to P. malariae.

Pathogenesis

The infectious cycle begins when sporozoites are inoculated into the bloodstream by an infected female Anopheles mosquito. The parasites first undergo hepatic replication (an asymptomatic phase), followed by release of merozoites that invade erythrocytes, contributing to anemia [3,26]. The ensuing inflammatory and immune responses underlie key clinical manifestations, including fever and splenomegaly [27,28]. Severe complications can occur, particularly with P. falciparum, including cerebral malaria, which is driven by sequestration of infected erythrocytes within the microvasculature, leading to capillary obstruction, hypoxia, edema, and coma [29].

PTM occurs because Plasmodium parasites reside within erythrocytes. Nevertheless, platelets, leukocytes, and even fresh (non-frozen) plasma may also transmit malaria when contaminated with residual infected erythrocytes [30]. It has been suggested that even ≤10 parasites in a donated unit may be infectious [31]. In addition, very low-level parasitemia (as low as 1 parasite/µL) can still deliver a clinically meaningful inoculum to the recipient [4].

Clinical Manifestations

Malaria most commonly presents with fever, sweats, headache, fatigue, nausea, and vomiting in uncomplicated cases, whereas severe disease may manifest with jaundice, seizures, coma, organ failure, and death. The most severe presentations are most often associated with Plasmodium falciparum infection [2]. Asymptomatic infection has also been described, defined as confirmed parasitemia in the absence of clinical symptoms; this is frequently attributed to partial immunity acquired after repeated exposure [32]. Importantly, asymptomatic carriers remain at risk of developing symptomatic disease and represent a major diagnostic and public health challenge because they can serve as a reservoir sustaining transmission in Africa and globally [2]. Compared with P. vivax, P. falciparum typically causes earlier and more severe anemia; P. vivax predominantly invades reticulocytes (young red blood cells), which may partially explain differences in hematologic severity [33].

PTM may be more severe than vector-borne malaria because parasites are introduced directly into the bloodstream, bypassing the hepatic stage that can contribute to early immune priming [23]. More severe courses have been reported in immunocompromised recipients [34]. In our review, all six patients had favorable outcomes following antimalarial therapy. The absence of reported PTM in some non-endemic African countries should be interpreted cautiously, as it likely reflects limited retrievable published case reports and/or underreporting rather than definitive evidence that PTM does not occur. In our setting, we occasionally encounter malaria cases despite Morocco no longer being endemic; however, no post-transfusion cases have been detected over the past 10 years.

Diagnosis

Malaria diagnosis is based on epidemiologic exposure, compatible symptoms, and laboratory testing [2]. For confirmation, microscopy of peripheral blood smears, including thick and thin films, remains the WHO-recommended gold standard, enabling species identification and estimation of parasite density, although accuracy depends on operator expertise and laboratory capacity [35]. Rapid diagnostic tests (RDTs) detect specific malaria antigens in blood and are particularly useful in resource-limited or remote settings, with generally high diagnostic performance [36]. Polymerase chain reaction (PCR) can provide sensitive species confirmation, and PCR and microscopy may be used for confirmation and follow-up when available [37].

Progress in Malaria Control Strategies

Approximately 95% of malaria cases and deaths occur in sub-Saharan Africa, where P. falciparum accounts for the majority of fatal outcomes [2]. Substantial progress has been made in malaria control in recent years, including in Africa, where most endemic regions are located. According to the WHO, several countries have recently been certified as malaria-free and are no longer considered endemic [17]. Table 3 summarizes African countries that have recently been declared malaria-free and their year of certification.

**Table 3:** African countries certified malaria-free by the World Health Organization (WHO) and year of certification. Source: [17].

Non-endemic country	Year of the WHO malaria-free certification
Cabo Verde	2024
Egypt	2024
Algeria	2019
Lesotho	2012
Libya	2012
Seychelles	2012
Tunisia	2012
Morocco	2010
Mauritius	1973

Prevention and Control Strategies

Multiple strategies are used to reduce malaria incidence by combining measures that limit transmission and protect high-risk groups, including pregnant women, children, and migrants. Public awareness and improved case detection through education and health promotion remain essential components of malaria control [2]. Vector control measures, including larval control and environmental management, can reduce mosquito breeding sites [38]. Insecticide-treated nets are associated with substantial reductions in malaria incidence and childhood mortality, and indoor residual spraying is linked to meaningful decreases in malaria risk [2].

Preventive measures also include chemoprophylaxis for travelers and seasonal malaria chemoprevention in endemic settings for eligible children and pregnant women during high-transmission seasons [39]. Common prophylactic options include sulfadoxine-pyrimethamine plus amodiaquine, atovaquone-proguanil, doxycycline, and mefloquine. Additional measures include protective clothing and the use of repellents. Malaria vaccines can provide partial protection that may wane over time [40].

Infectious Risk of Blood Transfusion: A Moroccan Perspective

Morocco defines donor selection criteria and contraindications and requires transfusion services to implement good transfusion practices covering the preparation, storage, labeling, and distribution of blood products, alongside mandatory biological testing for transfusion-transmissible infections and recipient immunohematologic testing. Morocco also mandates a hemovigilance system to monitor the blood supply chain and detect adverse transfusion reactions in recipients [41].

Screening step: The first step includes testing the donor and the donated blood. The donation is assessed for ABO hemolysins (anti-A/anti-B) and anti-erythrocyte antibodies, and screened for major transfusion-transmissible infections: human immunodeficiency virus (HIV) using HIV-1/2 antigen/antibody testing (Ag/Ab), hepatitis C virus (HCV) using anti-HCV, hepatitis B virus (HBV) using hepatitis B surface antigen (HBsAg), and syphilis using Venereal Disease Research Laboratory test (VDRL) and Treponema pallidum hemagglutination assay (TPHA). Alanine aminotransferase (ALAT) is also measured. If any screening result is positive, the donor is managed according to the specific finding. If ALAT is abnormal, the blood unit is destroyed (incinerated), and the donor is recalled for further evaluation [42].

Although transfusion-transmitted infections are rare, a non-negligible residual risk persists. Estimated risks include approximately 1 in 2 million for HIV, 1 in 300,000 for hepatitis B, and 1 in 1.5 million for hepatitis C; the risk for West Nile virus is approximately 1 in 350,000. In 2016, the Food and Drug Administration (FDA) began recommending Zika virus screening in blood centers because most infected individuals are asymptomatic. Malaria can also be transmitted through transfusion, resulting in a clinical malaria syndrome. Transmission of Plasmodium through transfusion is possible because parasites can remain viable under blood storage conditions, surviving at least 10 days and tolerating days to weeks at 2-6 °C in blood components [4]. In some countries, serologic tests are available for P. falciparum and P. vivax only. Countries such as the United Kingdom, France, and Australia report a low risk of PTM, supported in part by targeted approaches to identify malaria risk in donated blood [43].

Preventive Measures for Transfusion-Transmitted Infections in Morocco

Hemovigilance refers to a set of surveillance procedures spanning blood collection, transfusion, and post-transfusion follow-up of recipients, and it also includes epidemiologic monitoring of donors. Established in the early 1990s, its purpose is to collect and analyze adverse events related to the therapeutic use of blood products and to prevent their occurrence. It relies on donor medical assessment; good practices for collection, testing, preparation, storage, and transport; appropriate prescribing tailored to each recipient; communication between transfusion and care facilities; systematic transfusion and post-transfusion monitoring; and mandatory reporting of any unexpected reaction. In practice, hemovigilance is built around five key tools: traceability, prevention, incident reporting, patient information, and follow-up [44].

In non-endemic countries, mitigation has traditionally relied on donor questioning regarding travel, residence history, and possible malaria symptoms; policies have evolved to balance safety with donor loss. In parts of Europe, testing can shorten deferral, consistent with European regulations and guidance [45], whereas testing is not used in the United States/Canada, which rely on deferral alone. Although travelers from non-endemic areas comprise many *at-risk* donors, most PTM cases are linked to former residents of endemic areas who may carry chronic low-level parasitemia beyond historical deferral windows. This has prompted some authorities to require Plasmodium antibody testing for prior residents (and, in some settings, longer deferral and/or testing after return travel) [46]. Selective serologic screening has been reported as feasible and cost-effective, and several countries have implemented selective testing programs with very few subsequent PTM cases detected [47].

Additional risk-reduction approaches include leukoreduction filters, which may increase the removal of infected red blood cells [4]. Pathogen reduction technologies can further reduce PTM risk and are typically used for platelets and plasma, but they are not routinely applied to red blood cell components. Through this study, we aim to reinforce the importance of strict adherence to transfusion safety rules, particularly those addressing transmissible infections, and to emphasize maintaining clinical vigilance for post-transfusion infections caused by pathogens that are not systematically screened during routine transfusion testing. The growing number of malaria-free countries and declining case counts in some regions should not lead to complacency, particularly because many African countries remain high-burden settings, and transmission can be reintroduced through migration and international travel.

Treatment

Management of uncomplicated P. falciparum malaria relies primarily on combination therapies. Artemisinin-based regimens are standard (except in early pregnancy, when quinine plus clindamycin for seven days is recommended) [2]. Artemisinin-based combination therapies (ACTs) also treat mixed and many non-falciparum infections. Chloroquine may be used for uncomplicated P. vivax, P. ovale, and P. malariae in chloroquine-sensitive areas, and primaquine targets hepatic stages to prevent relapse [48].

Severe malaria carries a high mortality risk and requires rapid etiologic treatment plus aggressive supportive care, with intensive care unit transfer when needed. Intravenous artesunate is the first-line therapy, and pyronaridine phosphate is an alternative option where applicable [2].

Limitations of the Study

This systematic review is based on published case reports, series, and abstracts retrieved from accessible sources, so underreporting and publication bias are likely, and our findings cannot be used to estimate incidence. Many reports lacked key clinical, transfusion, donor, and outcome details; although 20 cases were identified overall, only six contained sufficient information for comprehensive extraction and comparison. Our search was conducted in English and French, and for each query, we screened the first 50 Google Scholar results; despite gray-literature searching and reference checking, relevant cases may have been missed. Finally, attribution of malaria to transfusion relied on the original reports’ laboratory confirmation in recipients and epidemiologic assessment supporting transfusion as the most plausible route, but donor testing and exclusion of alternative transmission routes were inconsistently documented, leaving a risk of misclassification

## Conclusions

In conclusion, PTM remains an uncommon but clinically important complication in non-endemic African countries, driven largely by asymptomatic low-level parasitemia in donors and amplified by diagnostic delays and reduced clinical suspicion outside endemic settings. Across all sources, we identified 20 reported cases, but only six were described in sufficient detail to allow synthesis, underscoring substantial underreporting and persistent gaps in case documentation and hemovigilance. Most well-characterized cases involved P. falciparum and occurred in vulnerable recipients, emphasizing the need for early smear-based testing and prompt treatment in any compatible post-transfusion fever. While current investigations in many countries rely primarily on donor questioning and deferral, targeted approaches, such as selective Plasmodium antibody testing for prior residents of endemic areas and risk-adapted testing strategies, may help reduce residual risk without excessive donor loss. Strengthening transfusion-chain quality assurance, improving systematic reporting, and maintaining clinical vigilance in malaria-free countries are essential to prevent avoidable morbidity and mortality from this largely preventable transfusion-transmitted infection.

## Appendices

Appendix A

**Table 4:** JBI Critical Appraisal Checklist (Case Reports) Y = Yes (reported), U = Unclear/Not reported in extracted data

Included record (country; year; ref)	Q1 Demographics clear	Q2 History clear	Q3 Presentation/clinical condition clear	Q4 Diagnostics clear	Q5 Intervention/treatment clear	Q6 Post-intervention outcome clear	Q7 Adverse events/harms reported	Q8 Takeaway lessons stated	Yes count
Morocco; 2024; [5]	Y	Y	Y	Y	Y	Y	U	U	6/8
Morocco; 2017; [6]	Y	Y	Y	Y	Y	Y	U	U	6/8
Morocco; 2011; [7]	U	Y	U	Y	U	U	U	U	2/8
Algeria; 2007; [9]	Y	Y	U	U	U	U	U	U	2/8
Tunisia; 2016; [11]	Y	Y	Y	Y	Y	Y	U	U	6/8
Mauritius; 1962; [13]	U	Y	Y	Y	Y	Y	U	U	5/8

Appendix B 

**Table 5:** Summary across items

JBI item	“Yes” among the 6 included records
Q1 Demographics clear	4/6
Q2 History clear	6/6
Q3 Presentation clear	4/6
Q4 Diagnostics clear	5/6
Q5 Intervention clear	4/6
Q6 Outcome/follow-up clear	4/6
Q7 Adverse events reported	0/6
Q8 Takeaway lessons stated	0/6
